# Leydig Cell Tumor Associated with Testicular Adrenal Rest Tumors in a Patient with Congenital Adrenal Hyperplasia due to 11**β**-Hydroxylase Deficiency

**DOI:** 10.1155/2012/648643

**Published:** 2012-02-12

**Authors:** Nadia Charfi, Mahdi Kamoun, Mouna Feki Mnif, Neila Mseddi, Fatma Mnif, Nozha Kallel, Basma Ben Naceur, Nabila Rekik, Hela Fourati, Emna Daoud, Zainab Mnif, Mourad Hadj Sliman, Tahia Sellami-Boudawara, Mohamed Abid

**Affiliations:** ^1^Endocrinology Department, Hedi Chaker Hospital, Sfax 3029, Tunisia; ^2^Department of Radiology, Hedi Chaker Hospital, Sfax 3029, Tunisia; ^3^Department of Urology, Habib Bourguiba Hospital, Sfax 3029, Tunisia; ^4^Anatomic Pathology Department, Habib Bourguiba Hospital, Sfax 3029, Tunisia

## Abstract

Congenital adrenal hyperplasia (CAH) describes a group of inherited autosomal recessive disorders characterized by enzyme defects in the steroidogenic pathways that lead to the biosynthesis of cortisol, aldosterone, and androgens. Chronic excessive adrenocorticotropic hormone (ACTH) stimulation may result in hyperplasia of ACTH-sensitive tissues in adrenal glands and other sites such as the testes, causing testicular masses known as testicular adrenal rest tumors (TARTs). Leydig cell tumors (LCTs) are make up a very small number of all testicular tumors and can be difficult to distinguish from TARTs. This distinction is interesting because LCTs and TARTs require different therapeutic approaches. Hereby, we present an unusual case of a 19-year-old patient with CAH due to 11*β*-hydroxylase deficiency, who presented with TARTs and an epididymal Leydig cell tumor.

## 1. Introduction

Congenital adrenal hyperplasia (CAH) describes a group of inherited autosomal recessive disorders characterized by enzyme defects in the steroidogenic pathways that lead to the biosynthesis of cortisol, aldosterone, and androgens.

Ninety percent of CAH cases have a defect in 21-alpha hydroxylase enzyme [1]. Steroid 11*β*-hydroxylase deficiency is the second most common cause of CAH, accounting for less than 5% of cases. It is characterised by the overproduction of adrenal androgens and deoxycorticosterone, leading to virilization of female fetuses, pseudoprecocious puberty in male infants, and hypertension with or without hypokalemia in both genders [2].

The rising of adrenocorticotropic hormone (ACTH) synthesis results in hyperplasia of ACTH-sensitive tissues in adrenal glands and other sites such as the testes, causing testicular masses known as testicular adrenal rest tumors (TARTs) [3]. It is extremely difficult to differentiate these masses from Leydig cell tumors (LCTs), which are the most common stromal testicular neoplasia [4, 5]. Both of these tumors could lead to precocious puberty and testicular masses [4]. Although the presentation of these two entities is the same, therapeutic approach is completely different. LCTs require surgical treatment, while most cases of TARTs respond to steroid suppressive therapy [4, 6].

Hereby, we present an unsual case of a 19-year-old patient with CAH due to 11*β*-hydroxylase deficiency, who presented with TARTs and an epididymal Leydig cell tumor.

## 2. Case Report

The patient was a known case of CAH due to 11*β*-hydroxylase deficiency since the age of 2-year old when he was evaluated for growth acceleration, advanced bone age, virilization, vomiting, diarrhea, generalized pigmentation, hypertension, and hypokalemia. Hormonal analyses were suggestive for the diagnosis of 11*β*-hydroxylase deficiency: 17-hydroxyprogesterone (17-OHP), 22 ng/mL (normal range 1.4–2.5); androstenedione, 11.9 ng/mL (normal range 0.9–2.2); 11-deoxycortisol: 69.6 ng/mL (normal <4) 8H Cortisol, 277 ng/mL (normal range from 43 to 250 ng/mL). His symptoms were controlled with 20 mg of hydrocortisone. He was irregular in followup and was not compliant with treatment. The patient was born of a fourth degree consanguineous marriage, and his family history was remarkable for early neonatal deaths.

At the age of 19 years, he was hospitalized for severe hypokalemia. On physical examination, he had a blood pressure of 180/110 mmHg, pulse rate of 70/min, temperature of 37°C, height of 163 cm (−2 SD), and weight of 58 kg (10th–25th percentile). A systemic melanoderma was noted. Genital examination demonstrated an enlarged right testis measuring 6.5 by 5.5 by 4.5 cm, and a 1.5-cm firm mass was noted in its upper pole. The left testis was not palpable in the scrotum or inguinal canal. The patient did not have any testicular discomfort or pain. He had no gynecomastia.

He had normal biochemistry values and a poorly compensated hormone profile: ACTH 2500 pg/mL (normal range 10–50 pg/mL); 17-OHP 17.5 ng/mL; androstenedione 19.9 ng/mL; dehydroepiandrosterone sulfate (DHEA-S) 90 *μ*g/mL (normal range 35–441); plasma deoxycorticosterone (DOC) 9648 ng/L (normal range: 40–170); testosterone 6.9 ng/mL (normal range 1.7–7.8). Tumor markers (*α*-fetoprotein, human chorionic gonadotropin, and lactate dehydrogenase) were negative for malignancy. Semen analysis showed severe oligo-astheno-teratospermia.

**Figure 1:**
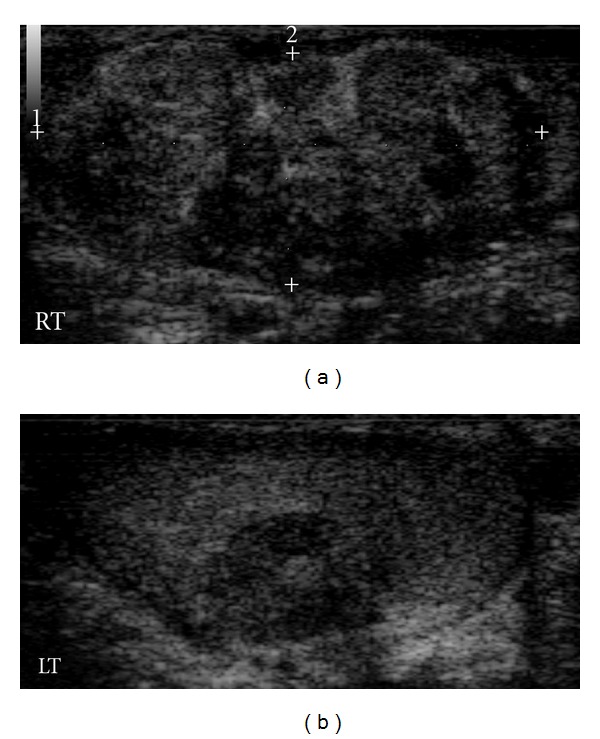
Longitudinal scrotal sonogram showing bilateral hypoechoic heterogeneous masses (RT: right testis, LT: left testis).

In testicular sonography, the right testis was present in the normal position and was enlarged (6.8 × 5.7 × 4.5 cm). However, the left testis was atrophic (3.5 × 2.8 × 1.5 cm) and ectopic, near the common iliac vessels. In addition, ultrasound scan of right testis revealed an hypoechoic heterogeneous mass within the lower pole measuring 4.8 × 3.8 cm in diameter. The left testis contained three round-to-oval hypoechoic heterogeneous lesions, 0.9–2.3 cm in diameter, located in the mediastinum testis (Figure 1). There was also evidence of a 1.5 cm hypoechoic mass of the right epididymis. Power Doppler ultrasound showed normal vascularity within the lesions.

**Figure 2:**
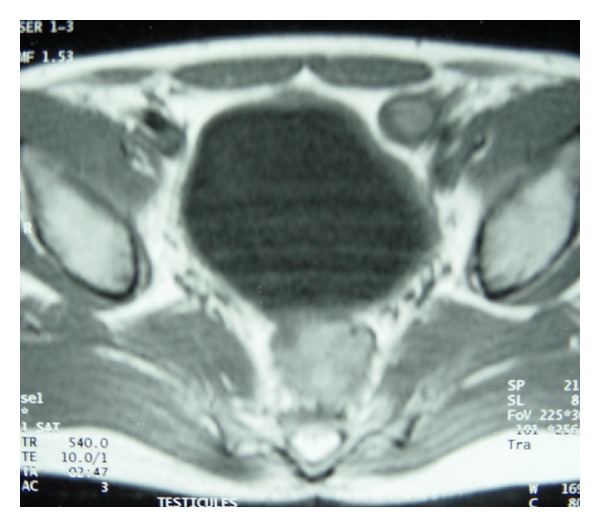
T1-weighted magnetic resonance image of the pelvis showing ectopic position of the left testis in front of the left commoun iliac vessels.

**Figure 3:**
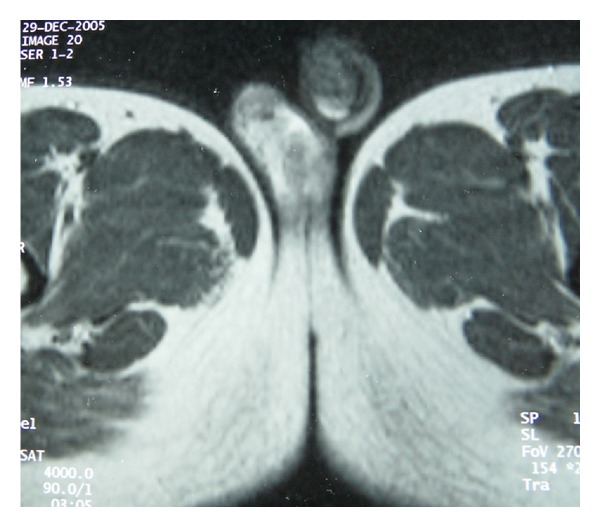
Testicular T2-weighted images showing bilateral hyperintense lesions with homogeneous enhancement.

**Figure 4:**
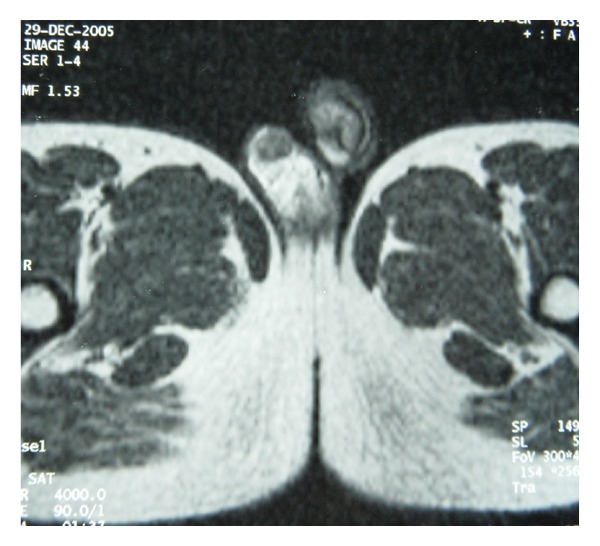
Testicular T2-weighted images showing hypointense lesion in the right epididymis, with central areas of high signal intensity.

The patient underwent a magnetic resonance imaging (MRI) examination. Coronal images of the pelvis confirmed the ectopic position of the left testis, in front of the left common iliac vessels (Figure 2). Bilateral testicular masses were found. The lesions were isointense on T1-weighted images and appeared hyperintense on T2-weighted images (Figure 3). They showed a homogeneous enhancement pattern. In addition, MRI study of the testis showed right epididymal mass exhibiting the following features: isointensity on T1-weighted images, marked enhancement on postcontrast images, and hypointensity on T2-weighted images with central areas of high signal intensity (Figure 4).

Four months after the administration of a stronger suppressive medical treatment with exogenous steroids (hydrocortisone 30 mg daily), a decrease in the size of right testis was noted (4 cm × 3 cm). Scrotal ultrasound showed reduction in the size of the right testicular mass (3.8 × 2.3 cm) but did not show any reduction in the size of the epididymal nodule. Hormone profile still remained poorly responsive to the compensation (17-OHP 8.1 ng/mL; testosterone: 9.1 ng/mL and 11-deoxycortisol: 11.3 ng/mL).

The patient was diagnosed as having bilateral testicular adrenal rest tumor associated with a suspicious-appearing mass in the right epididymis.

The patient underwent orchidopexy for the left ectopic testis, as well as an excisional biopsy of the right epididymal nodule. Bilateral biopsies of testicular parenchyma were also taken during the operation. 

**Figure 5:**
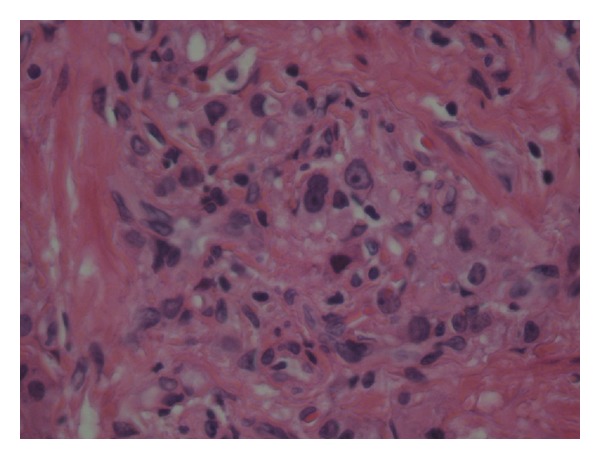
High power view of the epididymal tumor showing nests of polygonal cells with abundant granular eosinophilic cytoplasm (H and E, ×400). No necrosis or Reinke's crystal is seen.

**Figure 6:**
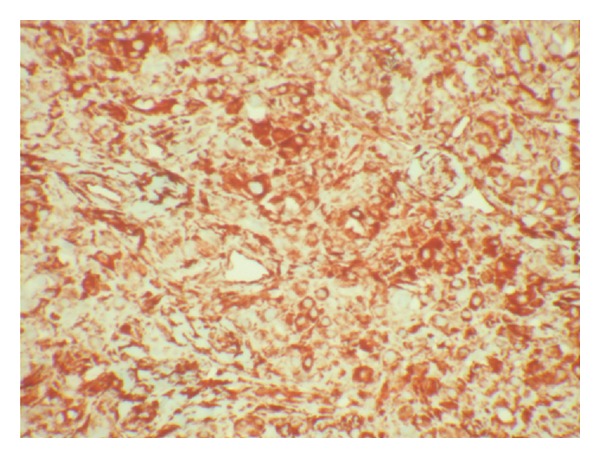
Immunoperoxidase stain for vimentin showing strong cytoplasmic positivity (IHC, ×100).

Pathologic examination of the right testis revealed that the epididymal nodule was well-circumscribed, encapsulated, firm, yellow, and measuring 15 mm in diameter. Microscopy showed that this nodule was composed of large polygonal cells with abundant eosinophilic, granular cytoplasm, and round, regular, vesicular nuclei with prominent nucleoli. The tumor cells were separated by an endocrinoid stroma. There was no evidence of infiltrating margins, necrosis, calcification, nuclear atypia, or vascular invasion. Both the lipochrome pigment and crystalloids of Reinke's were lacking (Figure 5). Immunohistochemistry showed widespread vimentin and alpha inhibin positivity (Figure 6). These features were consistent with epididymal Leydig cell tumor.

Testicular biopsy parenchyma showed a decrease in tubular diameter with peritubular fibrosis and tubular hyalinization. The lamina propria of the tubules was thickened. Extensive Leydig cell hyperplasia was seen in the interstitium, but no germ cells were present in seminiferous tubules.

After 24 months of followup, the patient is in good general health and continuing treatment with hydrocortisone. Scrotal ultrasound at the last visit showed bilateral multiple nodules stable in size. The patient is now preparing for partial orchiectomy.

## 3. Discussion

CAH patients with testicular enlargement present a difficult diagnostic dilemma. Tumors of adrenal rest tissue, LCTs, and Leydig cell hyperplasia are the primary etiologic considerations [7]. In this report, we describe a patient with CAH due to 11*β*-hydroxylase deficiency, presenting bilateral TARTs and epididymal Leydig cell tumor. To the best of our knowledge, such association has not been previously reported.

TARTs are one of the known complications of CAH. Their reported prevalence is up to to 94% of CAH adults, and they may already appear during childhood [8]. They probably develop from ectopic remnants of intratesticular adrenal tissue stimulated by ACTH hypersecretion [6]. Such tumors are always benign but compress the seminiferous tubules and lead to obstructive azoospermia, peritubular fibrosis, and irreversible damage to the surrounding testicular tissue, resulting in infertility. They may also have a paracrine effect via local steroid production which may be toxic to the Leydig cells and/or germ cells [6, 9].

LCTs are rare testicular tumors of the male gonadal interstitium, accounting for 1–3% of all testicular neoplasms. These tumors are most common in prepubertal boys aged 5–10 years and in adults aged 30–60 years [10]. Approximately 10% of LCTs are malignant, and this variant occurs exclusively in adults [4, 11].

The etiology of LCTs remains unknown. Unlike germ cell testicular tumors, Leydig cell neoplasms are not associated with cryptorchidism. It is thought that an endocrine role may contribute to the development of these tumors. The disruption of the hypothalamic-pituitary testicular axis leading to excessive stimulation of Leydig cells by excess luteinizing hormone may induce Leydig cells oncogenesis [12]. High estrogen levels also could play a role in the neoplastic transformation of Leydig cells [13].

LCTs are frequently hormonally active, leading to feminizing or virilizing syndromes. These tumors were once managed primarily with radical orchiectomy. However, the experience with conservative procedures has been growing [14].

The occurrence of leydigioma in CAH patients is exceptional, and only six cases of Leydig-cell tumour associated with CAH have been reported so far [3, 15–18]. These reports have raised concerns about the distinction of LCT from TARTs. Such distinction is interesting, given that there is a different approach to each pathology; LCTs require surgical treatment, while most cases of TARTs respond to medical treatment [4, 6]. To differentiate between the two syndromes, some features have been reported, even if there are no pathognomonic characteristics [19]. TARTs are usually bilateral, painful, and found typically adjacent to hilum of the testis. Indeed, they generally regress with corticosteroid replacement [4]. In contrast, LCTs are usually unilateral, painless, unresponsive to ACTH suppression therapy, and located in the interstitial tissue of testis next to the seminiferous tubules. There are no biochemical markers enabling differentiation between the 2 syndromes although high level of plasma testosterone may be suggestive of LCTs [20]. Histologically, there is a strict resemblance between the Leydig cell tumor and adrenocortical cells typical of CAH testicular nodules. Cytoplasmic rod-like crystalloids called Reinke crystals are characteristic of the Leydig cell tumor, but they are not positive in 60% of patients with LCT [21]. In addition, Inhibin A could be a sensitive and a specific immunohistochemical marker for LCTs [22].

In our patient, unilaterality, the lack of response to glucocorticoid therapy and immunohistological features were suggestive of the diagnosis of a Leydig cell tumor. In addition, imaging characteristics argue also for this diagnosis. Indeed, as seen in our patient, LCTs are typically isointense on T1-weighted, -showed enhancement and are hypointense on T2 weighted with central areas of increased signal [23].

## 4. Conclusion

Our report illustrates an unusual association of epididymal Leydig cell tumor with TARTs in a patient with CAH due to 11*β*-hydroxylase deficiency. The distinction between these two tumors is often difficult, but interesting as treatment modalities are different. CAH patients with testicular tumor need cautious approach during investigations to avoid inappropriate orchiectomies.
